# Inorganic Phosphate in the Pathogenesis of Pregnancy-Related Complications

**DOI:** 10.3390/ijms21155283

**Published:** 2020-07-25

**Authors:** Ana Correia-Branco, Monica P. Rincon, Leonardo M. Pereira, Mary C. Wallingford

**Affiliations:** 1 Mother Infant Research Institute, Tufts Medical Center, 800 Washington Street, Boston, MA 02111, USA; ana.clmc.branco@gmail.com; 2 Maternal Fetal Medicine, Oregon Health Science Center, Mailcode L-458, 3181 SW Sam Jackson Park Road, Portland, OR 97219, USA; rincon@ohsu.edu (M.P.R.); pereiral@ohsu.edu (L.M.P.)

**Keywords:** inorganic phosphate, preeclampsia, diabetes mellitus, pregnancy, placental calcification

## Abstract

Inorganic phosphate (P_i_) is an essential nutrient that fulfills critical roles in human health. It enables skeletal ossification, supports cellular structure and organelle function, and serves key biochemical roles in energetics and molecular signaling. P_i_ homeostasis is modulated through diet, intestinal uptake, renal reabsorption, and mobilization of stores in bone and extracellular compartments. Disrupted P_i_ homeostasis is associated with phosphate wasting, mineral and bone disorders, and vascular calcification. Mechanisms of Pi homeostasis in pregnancy remain incompletely understood. The study presented herein examined biological fluid Pi characteristics over the course of gestation. Correlations with gestation age, pregnancy number, preterm birth, preeclampsia, diabetes mellitus, and placental calcification were evaluated during the last trimester. The results support that maternal urinary P_i_ levels increased during the third trimester of pregnancy. Reduced levels were observed with previous pregnancy. Amniotic fluid P_i_ levels decreased with gestation while low second trimester levels associated with preterm birth. No significant difference in urinary P_i_ levels was observed between preeclampsia and controls (8.50 ± 2.74 vs. 11.52 ± 2.90 mmol/L). Moreover, increased maternal urinary P_i_ was associated with preexisting diabetes mellitus in preeclampsia. Potential confounding factors in this study are maternal age at delivery and body mass index (BMI)—information which we do not have access to for this cohort. In conclusion, P_i_ levels provide clinical information regarding the pathogenesis of pregnancy-related complications, supporting that phosphate should be examined more closely and in larger populations.

## 1. Introduction

Phosphorus is an essential micronutrient that serves as key biochemical roles in cellular structure, organelle function, energetics and molecular signaling, and skeletal ossification. Phosphorus is a primary component of hydroxyapatite crystals, which are minerals that reinforce bone strength [1,2]. Elemental phosphorus circulates in the blood in the form of phosphoric acid (PO_4_), also termed inorganic phosphate or phosphate (P_i_). P_i_ homeostasis is modulated through diet, intestinal uptake, renal reabsorption, and mobilization of stores in bone and extracellular compartments. Disrupted P_i_ homeostasis is associated with P_i_ wasting, refeeding syndrome, chronic kidney disease (CKD) mineral and bone mineralization disorders (BMD), and medial vascular calcification. P_i_ transport into cells (symport) occurs against a 100-fold concentration gradient via transmembrane phosphate transporter proteins, such as the type III sodium-dependent phosphate transporters Slc20a1 (PiT-1) and Slc20a2 (PiT-2). In early preeclampsia (PE), placental expression of the sodium-dependent phosphate transporters Slc20a1 and Slc20a2 is highly reduced; yet in late PE, Slc20a2 is significantly increased [3]. The cause for these alterations remains unknown. In the nongravid state, P_i_ homeostasis is regulated through multi-system endocrine signaling in conjunction with calcium via systemic activity of phosphatonins including parathyroid hormone (PTH), vitamin D (VD), and fibroblast growth factor 23 (FGF23). The parathyroid gland responds to decreased calcium levels and regulates PTH secretion which increases blood calcium levels by stimulating release of calcium and P_i_ from bone, as well as altering calcium reabsorption, P_i_ excretion, and VD production by the kidney, which in turn increases intestinal reabsorption [4,5,6,7,8]. FGF23 signaling downregulates P_i_ transporter gene expression and inhibits production of VD and PTH [4,5]. During fetal development, placental mineral transport and micronutrient accretion is regulated in part by PTH-related protein (PTHrP) and possibly PTH, but not by calcitriol, FGF23, calcitonin, or the sex steroids [5]. In the fetus, fetal kidneys and fetal membranes contribute to production and regulation of amniotic fluid and P_i_ can undergo several routes [5,9]. At least five pathways of exchange have been identified between the amniotic space and the surrounding tissues, indicating multiple routes by which P_i_ can be exchanged between amniotic fluid and the developing fetus, where the large majority of P_i_ undergoes accretion and plays a crucial role in skeletal ossification during the latter half of pregnancy [9]. In nonpathological conditions, fetal serum P_i_ levels are maintained approximately 0.5 mmol/L higher than in the mother [5].

PE is a complex and pregnancy-specific hypertensive syndrome. It is reported that women who have developed PE in pregnancy are at increased risk of cardiovascular disease, coronary artery calcification, stroke, and chronic kidney disease later life [10,11,12,13,14]. PE is characterized by hypertension and proteinuria after 20 weeks’ gestation, and is frequently associated with placental dysfunction, although the etiology remains unknown at the molecular level [15,16]. PE affects 3–5% of pregnancies worldwide and is one of the leading causes of maternal and fetal morbidity and mortality [16,17,18]. The standard features of PE are new-onset hypertension (defined as a systolic blood pressure of 140 mm Hg or more, or a diastolic blood pressure of 90 mm Hg or more on two occasions at least 4 h apart after 20 weeks of gestation; or systolic blood pressure of 160 mm Hg or more, or a diastolic blood pressure of 110 mm Hg or more on two occasions at least 4 h apart after 20 weeks of gestation) and proteinuria (300 mg or higher in a 24 h urine specimen) [16]. Pregnancy adaptations change maternal bone metabolism, resulting in an uncoupling of mineral resorption and deposition rates to a state of high bone turnover to support the skeletal bone growth requirements of the fetus [19]. Studies show that bone turnover biochemical markers increase in pregnancies complicated by preeclampsia [20]. Interestingly enough, although maternal bone mineral density (BMD) is unaffected by preeclampsia [21,22], adult offspring who were previously exposed to maternal preeclampsia in the womb present higher BMD levels than those not exposed [23,24].

High levels of dietary Pi consumption and high circulating levels of Pi induce ectopic medial vascular calcification, the deposition of calcium-phosphate mineral in the smooth muscle cell layer of blood vessels. Vascular calcification requires phosphate for the chemical foundation of the calcium-phosphate mineral, in the form of hydroxyapatite, and high Pi levels induce pathological expression of osteochondrogenic proteins by smooth muscle cells [25,26,27]. High extracellular phosphate has been described to impose a significant cardiovascular risk through promoting vascular calcification, an independent risk factor for cardiovascular morbidity [26]. Ectopic calcification can occur in the placenta tissue and is frequently observed in pregnancy. However, clinical research on the association between placental calcification and clinical outcomes and acute disease such as PE is limited, the etiology of placental calcification remains undefined, and the clinical significance of placental calcification remains unclear [18,28,29,30]. Therefore, the aim of this research was to examine biological fluid (maternal urine, amniotic fluid) P_i_ characteristics over the course of gestation and to evaluate correlation with gestational age at delivery, pregnancy number, preterm birth, preeclampsia, diabetes mellitus, and placental calcification.

## 2. Materials and Methods

### 2.1. Nonhuman Subjects Research

Ethical review for the enclosed de-identified amniotic fluid, urine, and placenta analyses were performed by the University of Washington Human Subjects Division. Subcommittee E/B determined that this activity does not meet the federal regulatory definition of “human subjects” research under 45 CFR 46.102. Application numbers are as follows: Non-Identifiable HSD #47984 (placenta and urine specimens) and HSD #51806 (amniotic fluid specimens).

### 2.2. GAPPS Cohort Samples

Maternal urine and placental tissue section samples from 9 normotensive pregnant women and 7 preeclamptic women were purchased from the Global Alliance for the Prevention of Prematurity and Stillbirth (GAPPS). Preeclampsia was defined by standard clinical criteria [16]. Gestational ages were between 28.7 and 33 weeks. Samples were coded by GAPPS without any patient identifiers and analyzed without knowledge of the disease status. Specimens from participants with a history of smoking were excluded from this study. Participants were characterized with preexisting pregnancies and divided in two groups as follows: first pregnancy and second pregnancy. Participants were characterized with preexisting diabetes mellitus, which included Type I, Type II and Gestational Diabetes Mellitus, and divided in two groups as follows: normal glucose tolerance (NGT) and diabetes mellitus. Maternal urine and amniotic fluid aliquots were stored at −80 °C until analysis.

### 2.3. OHSU Amniotic Fluid Samples

De-identified amniotic fluid samples were obtained from OHSU (MTA-OUT16-095). A total of 11 s trimester and 9 third trimester samples were obtained. Gestation age of delivery (GAD) ranged between 25 and 39 weeks on average. Gestation age of sample collection (GASC) ranged between 17 and 35 weeks on average. We tested whether amniotic fluid phosphate levels were an indicator for preterm or early pre-term birth for second and third trimester of pregnancy.

### 2.4. P_i_ Quantification

Maternal urine P_i_ levels were determined with the Phosphate Assay Kit (Sigma Aldrich, St. Louis, MO, USA; MAK308) and amniotic fluid P_i_ levels were determined with the QuantiChrom Phosphate Assay Kit (Bioassays Systems, Hayward, CA, USA; DIPI500) according to the manufacturer’s instructions.

### 2.5. Methods for Von Kossa Staining and Imaging of Placenta Tissue

The von Kossa staining was performed as previously described in Speer et al. 2009 [31] with the inclusion of an optimally reduced 22 min von Kossa treatment. Placental tissue section from 8 normotensive pregnant women and 8 preeclamptic women were stained, and images of mounted sections were acquired on a Keyence BZ-X800 microscope using the Keyence BZ-X software (Keyence, Ozaka, Japan) at 4× magnification. File names were coded. Individual calcified lesions were imaged on a Nikon E800 Upright Microscope (Nikon Corp., Tokyo, Japan) at 2.5 and 10× magnification and file names were coded. Semi-quantitative analysis of calcified lesions was performed on all tissue sections using ImageJ/Fiji (NIH, Bethesda, MD, USA) histomorphometry software.

### 2.6. Statistical Analysis of Experimental Data

The following statistical tests were used to analyze quantitative data in GraphPad Prism 6 software for Windows (GraphPad Software Inc., La Jolla, CA, USA). For comparison of two groups, a *p*-value was determined by a two-tailed Student’s T-test with unequal variance. For comparison of three or more groups, a *p*-value was determined by a Welch’s T-test with unequal variance. Slope calculations were obtained by Deming (model II) linear regression.

## 3. Results

### 3.1. Clinical Characteristics

**Figure 1:**
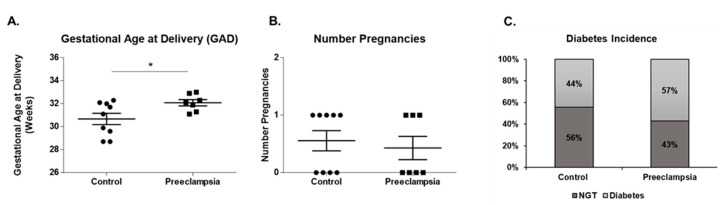
Clinical characteristics of cohort and association with preeclampsia. Clinical characteristics of cohort (control, *n* = 9; preeclampsia, *n* = 7). We observed that gestational age at delivery (GAD) was increased in the Preeclampsia group (**A**). We did not observe differences on the preexistence of pregnancies (**B**) and diabetes incidence (**C**) in both Control and Preeclampsia groups. Symbols: ● Control; ▪ Preeclampsia; * *p* ≤ 0.05. Abbreviations: GAD: Gestational Age at Delivery; NGT: normal glucose tolerance.

Participant characteristics are shown in Figure 1. The gestational ages at delivery (GAD) is statistically significantly higher in the preeclampsia group compared to the normotensive group (32.08 ± 0.27 and 30.68 ± 0.49 weeks; *p* = 0.0375; Figure 1A). A statistically significant association was not observed between preexisting pregnancies and normotensive or preeclampsia groups (0.56 ± 0.18 and 0.43 ± 0.20; *p* = 0.6420; Figure 1B), or preexisting diabetes mellitus (100% ± 40% and 129% ± 45%; *p* = 0.6420; Figure 1C).

### 3.2. Increased Maternal Urine P_i_ Levels are Associated with Preeclampsia

**Figure 2:**
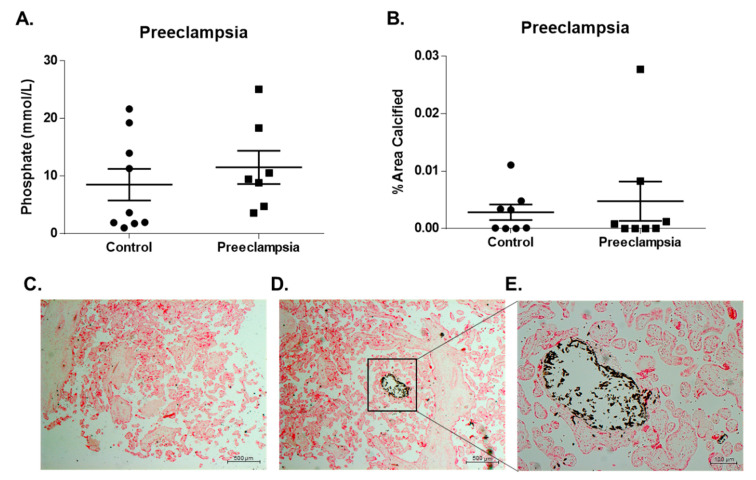
Maternal urine inorganic phosphate (P_i_) levels association with preeclampsia and placental calcification. Phosphate levels were determined for maternal urine (control, *n* = 9; preeclampsia, *n* = 7), and we observed a trend of increased phosphate levels with preeclampsia (**A**). urine (control, *n* = 9; preeclampsia, *n* = 7), and we observed a trend of increased phosphate levels with preeclampsia (**A**). Placental calcification (% Area Calcified) was determined for placental tissue sections (control, *n* = 8; preeclampsia, *n* = 8), and we observed a trend of increased placental calcification with preeclampsia (**B**). Images of von Kossa staining of a placental tissue section of preeclampsia donor identify negative (**C**) and positive regions (**D,E**) (black deposits; **C** and **D** magnification ×2.5, **E** magnification ×10). Box in (**D**) correspond to region imaged in (**E**). The distribution of the crystal deposits suggested a formation of calcium-phosphate mineral deposition at chorionic villi. Symbols: ● Control; ▪ Preeclampsia; * *p* ≤ 0.05.

Decreased levels of P_i_ have been described in the maternal urine of preeclampsia patients [32]. We quantified P_i_ excretion in maternal urine from normotensive and preeclampsia groups. The average P_i_ maternal urine level was higher in the preeclampsia group (8.50 ± 2.74 vs. 11.52 ± 2.90 mmol/L; *p* = 0.4653), but this difference was not statistically significant (Figure 2A). Ectopic vascular calcification has been previously associated with preeclampsia [22,33]. von Kossa staining was used to detect calcified lesions on placental tissue slides of the preeclampsia group studied (Figure 2B–E). The average % Area Calcified was higher in the preeclampsia group (0.0028 ± 0.0014 vs. 0.0048 ± 0.0034; *p* = 0.6117), but this difference was not statistically significant (Figure 2B). The distribution of the crystal deposits (black deposits) suggested a formation of calcium-phosphate deposits in chorionic villi (Figure 2D–E).

### 3.3. Maternal Urine P_i_ Levels Increase during Gestational Age

**Figure 3:**
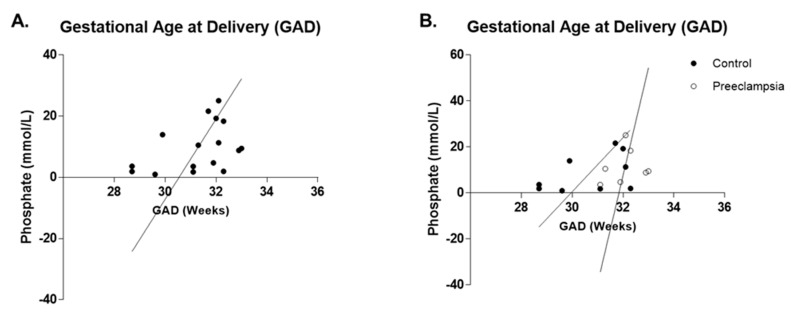
Maternal urine inorganic phosphate (P_i_) levels association with gestational age at delivery (GAD). Phosphate levels were determined for maternal urine (control, *n* = 9; preeclampsia, *n* = 7). We observed that phosphate levels increase with time during third trimester of pregnancy, in all cohort (**A**) and in both Control and Preeclampsia groups (**B**). Abbreviations: GAD: Gestational Age at Delivery.

Maternal urine P_i_ levels were determined to assess P_i_ variations over the course of human gestation. We observed that P_i_ excretion increased between weeks 28–34 during third trimester of pregnancy in all cohorts (Figure 3A), and in both control and preeclampsia groups (Figure 3B).

### 3.4. Maternal Urine P_i_ Levels Altered with Number of Pregnancies and with Diabetes and Preeclampsia

**Figure 4:**
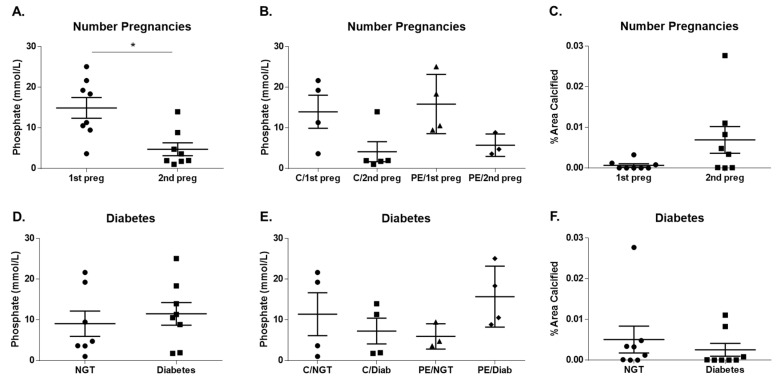
Maternal urine inorganic phosphate (P_i_) levels and placental calcification association with preexisting pregnancies, diabetes and preeclampsia. Phosphate levels were determined for maternal urine (control, *n* = 9; preeclampsia, *n* = 7). We observed that phosphate levels decrease with preexisting pregnancies in all cohorts (**A**). We observed a trend of reduced phosphate levels with preexisting pregnancies in both the Control and Preeclampsia groups (**B**). We observed a trend of increased phosphate levels with preexistence of diabetes in all cohorts (**D**) and Preeclampsia groups (**E**). Placental calcification (% Area Calcified) was determined for placental tissue sections (control, *n* = 8; preeclampsia, *n* = 8), and we observed a trend of increased placental calcification with preexistence of previous pregnancies in all cohorts (**C**) and decreased with preexistence of diabetes (**F**). Symbols: * *p* ≤ 0.05. Abbreviations: diab: diabetes; NGT: normal glucose tolerance; PE: preeclampsia; preg: pregnancy.

We observed that P_i_ levels decreased significantly on maternal urine with increased number of pregnancies (14.92 ± 2.57 vs. 4.72 ± 1.59 mmol/L; *p* = 0.0045; Figure 4A). This trend of decreased P_i_ excretion in a second pregnancy for both normotensive and preeclampsia groups (Figure 4B), points to a putative decrease in bioavailability of phosphate with a second pregnancy. Furthermore, we observe a trend of increased average % Area Calcified in second pregnancies for both normotensive and preeclampsia groups (0.0007 ± 0.0004 vs. 0.0069 ± 0.0033; *p* = 0.0805; Figure 4C). Association of maternal urinary P_i_ levels with preexisting diabetes mellitus (100% ± 35% vs. 141% ± 34% mmol/L; *p* = 0.4180; Figure 4D) and preeclampsia (Figure 4E) was evaluated, and indicated that diabetes dysregulates Pi excretion during pregnancy, and this is exacerbated with superimposed preeclampsia. In contrast, a modest trend of decreased average % Area Calcified was observed with preexisting diabetes for both normotensive and preeclampsia groups (0.0051 ± 0.0033 vs. 0.0025 ± 0.0016; *p* = 0.5005; Figure 4F).

### 3.5. Amniotic Fluid P_i_ Levels Decrease during Gestational Age

**Figure 5:**
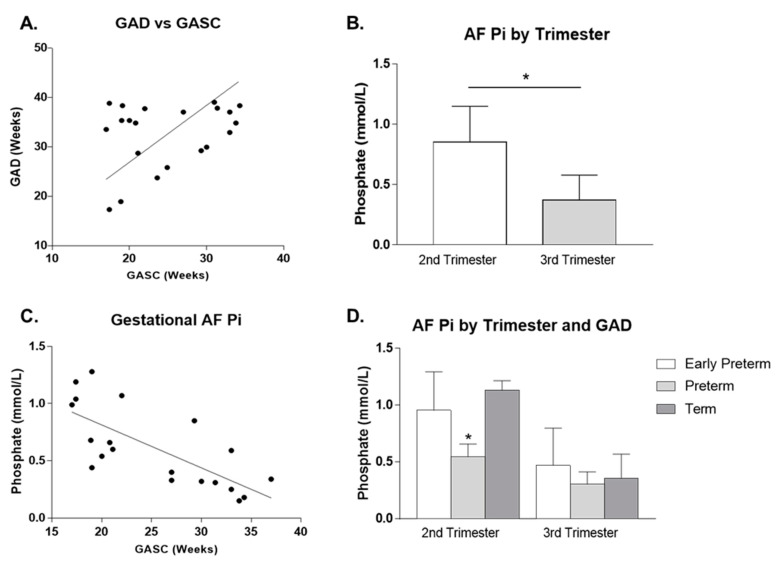
Amniotic fluid (AF) inorganic phosphate (P_i_) as an indicator of gestational age. Phosphate levels were determined with amniotic fluid collected between 17 and 35 weeks of pregnancy and we tested whether phosphate was an indicator for preterm birth (**A**). We observed that phosphate levels decrease with time during pregnancy (**B, C**) and an association between low second trimester levels and preterm birth (**D**). Symbols: * *p* ≤ 0.05. Abbreviations: AF: amniotic fluid; GAD: gestational age at delivery; GASC: gestational age at sample collection; Pi: inorganic phosphate.

Amniotic fluid Pi levels were determined to assess P_i_ variations over the course of human gestation. The gestational age at sample collection (GASC), between 17 and 35 weeks of pregnancy, overlaps the gestational age at delivery (GAD) 25 and 39 weeks (Figure 5A), which includes both second and third trimester of pregnancy. We observed that P_i_ levels decrease with time during pregnancy (Figure 5C–D), in accordance with a previous study ran between 16–26 weeks [34]. Lastly, we tested whether P_i_ was an indicator of preterm birth and observed an association between low second trimester phosphate amniotic fluid levels and preterm birth, supporting that P_i_ should be examined more closely and in a larger population (Figure 5D).

## 4. Discussion

Phosphorus is an essential micronutrient involved in a number of required biological processes. The purpose of this study was to characterize phosphate level dynamics over the course of gestation in maternal and fetal biological fluids. Hypophosphaturia is an important feature of severe preeclampsia that may be indirectly related to altered renal function [32]. The correlation with gestational age at delivery (GAD), pregnancy number, preterm birth, preeclampsia, diabetes mellitus, and placental calcification was evaluated. Potential confounding factors include maternal age at delivery and body mass index (BMI)—information which we do not have access to. Furthermore, the average GAD is higher for the preeclampsia group compared to the normotensive group, whilst no difference was observed regarding preexisting pregnancies and diabetes mellitus.

Maternal phosphate excretion increases during pregnancy, with maximum phosphate urine levels observed during the third trimester [35]. Similar to these findings, our study shows that maternal urine P_i_ levels increased linearly between weeks 28–34 during the third trimester of pregnancy in all cohorts, including normotensive and preeclampsia groups. This supports that during pregnancy, decreased vascular resistance may induce adaptions, sustained by nitric oxide production, which results in the expansion of plasma volume by stimulating renal sodium and water retention and contributes to higher glomerular filtration rate (GFR) [36]. Renal excretion of both calcium and phosphate is decreased in preeclamptic women compared to normotensive women [32,37,38,39], as a possible result of a compensatory mechanism of decreased renal filtration rate and increased tubular reabsorption of calcium and phosphate in toxemia [32,39]. However, we observed a tendency of increased maternal urinary phosphate excretion in the preeclampsia group. The GAD in the preeclampsia is modestly higher in the preeclampsia group, which may contribute to this difference. Ectopic vascular calcification has also been previously associated with preeclampsia [22,33]. Indeed, we were able to detect calcified lesions on placental chorionic villi tissue slides of the preeclampsia group. We observed a trend of increased placental calcification on the preeclampsia group studied; however, by this method, there was no significant association in this cohort.

Phosphate metabolism is regulated by bone-derived FGF-23 and PTH, which influence the renal production and circulating concentrations of the active metabolite of VD, 1,25-hydroxyvitamin D [1,25(OH)D], which affects bone metabolism, intestinal absorption of calcium and phosphorus, and hypertension and vascular calcification [40]. Elevated serum phosphate has an inhibitory effect on the renal activation of 25-hydroxyvitamin D [25(OH)D] and 1,25-hydroxyvitamin D [1,25(OH)D], and lower concentrations of 1,25(OH)D have been associated with adverse cardiovascular outcomes [40]. During pregnancy, maternal serum levels of 1,25(OH)D increase up to twofold starting at 10–12 weeks of gestation and reaching a maximum in the third trimester [41,42]. As reviewed elsewhere [42], preeclamptic women are described to present lower circulating 25(OH)D3 levels than normotensive pregnant women, and VD supplementation showed promise for ameliorating the risk of PE. Contrary to the expected, we report an increase in maternal phosphaturia associated with preexisting diabetes mellitus alone. We also found that maternal urine P_i_ levels are higher in association with preexisting diabetes and preeclampsia compared to diabetes and normotensive participants. Moreover, placental calcification was shown to decrease with preexisting diabetes. Dietary intake may influence these differences, as Mancini FR et al. [43] reported a linear association between increasing dietary phosphorus intake, and the risk for type 2 diabetes in women.

To the best of our knowledge, the present study is the first to demonstrate an association between the number of pregnancies and maternal urine phosphate levels. Indeed, we observed that maternal urine P_i_ levels decreased with a second pregnancy, which was consistent for both normotensive and preeclampsia groups, pointing to a putative decrease in the bioavailability of phosphate with a second pregnancy. However, mothers with preexisting pregnancies may present higher maternal age at delivery. It is well accepted that advanced maternal age is a risk factor for perinatal and neonatal outcomes, gestational diabetes mellitus, gestational hypertension, preeclampsia, small for gestational age infants, spontaneous late preterm delivery, and cesarean section [44]. Furthermore, we observed a trend of increased placental calcification with the number of pregnancies; however, there was no significant association in this cohort.

Lastly, we tested whether phosphate in amniotic fluid was an indicator of preterm birth and its variation over the course of gestation. We observed that amniotic fluid phosphate levels decreased with gestation while low second trimester levels were associated with preterm birth, which suggested that amniotic fluid may be useful as a marker of gestational age. These observations are in conformity with results previously reported [34,45,46], that demonstrate that, during pregnancy, concentrations of phosphate [34,45,46] and calcium [45] decreased, and levels of uric acid [34,45] and creatinine [45,46] increased. This is consistent with the increasing fetal glomerular filtration rate and progressive maturation of tubular function, thus more micronutrients are resorbed at fetal tubules and, consequently, less will be excreted to the amniotic fluid [34,45,46].

During pregnancy, maternal bone mineral density (BMD) and bone resorption markers are modestly increased [21,22,42,47]. With increased number of pregnancies, we hypothesize that the BMD and phosphorus and calcium bioavailability is reduced. However, the information regarding maternal pre-pregnancy BMI as an indicator of maternal nutritional status was not available. Potential limitations to this study should be considered and addressed through future studies that build on these findings to evaluate candidate modifiers, such as maternal age at delivery, maternal body mass index (BMI), biological levels of calcium, VD, PTH, genetics and lifestyle. Future work will evaluate the relationship between phosphate dysregulation in preeclampsia, diabetes, maternal age, and number of pregnancies through the analysis of the tissue-specific and cell-specific nature of calcified lesions; expression of placental phosphate transporter expression levels (Slc20a1 and Slc20a2) and osteochondrogenic factors; maternal urinary calcium and phosphate excretion; and maternal age. In conclusion, phosphate levels provide clinical information regarding temporal fluctuation of maternal–fetal phosphate homeostasis in pregnancy-related complications, supporting that phosphate should be examined more closely and in larger populations.

## Abbreviations

1:25(OH)D	1,25-hydroxyvitamin D
BMD	Bone mineralization disorders
CKD	Chronic kidney disease
FGF23	Fibroblast growth factor 23
GAD	Gestation age of delivery
GASC	Gestation age of sample collection
GFR	Glomerular filtration rate
NGT	Normal glucose tolerance
PE	Preeclampsia
P_i_	Inorganic phosphate
PO_4_	Phosphoric acid
PTH	Parathyroid hormone
PTHrP	PTH-related protein
Slc20a1(PiT-1)/Slc20a2 (PiT-2)	Sodium-dependent phosphate transporters
VD	Vitamin D
